# Accelerated Healing of Diabetic Wounds Treated with L-Glutamic acid Loaded Hydrogels Through Enhanced Collagen Deposition and Angiogenesis: An *In Vivo* Study

**DOI:** 10.1038/s41598-017-10882-1

**Published:** 2017-09-06

**Authors:** Ponrasu Thangavel, Balaji Ramachandran, Sudip Chakraborty, Ramya Kannan, Suguna Lonchin, Vignesh Muthuvijayan

**Affiliations:** 1 Department of Biotechnology, Bhupat and Jyoti Mehta School of Biosciences, Indian Institute of Technology Madras, Chennai, 600036 India; 2 Department of Chemistry, Indian Institute of Technology Madras, Chennai, 600036 India; 3 Department of Biochemistry and Biotechnology, CSIR-Central Leather Research Institute, Adyar, Chennai, 600020 India

## Abstract

We have developed L-glutamic acid (LG) loaded chitosan (CS) hydrogels to treat diabetic wounds. Although literature reports wound healing effects of poly(glutamic acid)-based materials, there are no studies on the potential of L-glutamic acid in treating diabetic wounds. As LG is a direct precursor for proline synthesis, which is crucial for collagen synthesis, we have prepared CS + LG hydrogels to accelerate diabetic wound healing. Physiochemical properties of the CS + LG hydrogels showed good swelling, thermal stability, smooth surface morphology, and controlled biodegradation. The addition of LG to CS hydrogels did not alter their biocompatibility significantly. CS + LG hydrogel treatment showed rapid wound contraction compared to control and chitosan hydrogel. Period of epithelialization is significantly reduced in CS + LG hydrogel treated wounds (16 days) compared to CS hydrogel (20 days), and control (26 days). Collagen synthesis and crosslinking are also significantly improved in CS + LG hydrogel treated diabetic rats. Histopathology and immunohistochemistry results revealed that the CS + LG hydrogel dressing accelerated vascularization and macrophage recruitment to enhance diabetic wound healing. These results demonstrate that incorporation of LG can improve collagen deposition, and vascularization, and aid in faster tissue regeneration. Therefore, CS + LG hydrogels could be an effective wound dressing used to treat diabetic wounds.

## Introduction

Globally, diabetes mellitus (DM) is one of the major health concerns with increasing prevalence. DM is a common chronic endocrine disorder that primarily results in hyperglycemia due to the absence or insufficiency and resistance to insulin^1, 2^. Wounds in diabetic patients are slow to heal and persist for a few months under proper wound care and management. Wound healing is a multifaceted and overlapping process that follows a systematic sequence. There are three main stages involved in the process of wound healing: inflammation, proliferation, and remodeling. Inflammation follows hemostasis (blood clotting) and can last for up to 6 days^3^. During the proliferative phase, new blood vessels are formed by angiogenesis, collagen is synthesized and deposited. This further leads to the formation of granulation tissue, epithelialization, and wound contraction^4^. Tissue remodeling phase starts at the end to replace the granulation tissue with a scar tissue, which takes approximately 3 to 6 weeks^3^. In the case of diabetic wounds, the inflammation phase is prolonged and the proliferative phase fails to begin. This leads to chronic unhealed wounds^5^. Thus, diabetic wound healing is a serious challenge in clinical practice^4^. Pathophysiology of impaired diabetic wound healing is still unclear. It is presumed that delayed healing is due to the persistence of prolonged inflammation, and an inadequate angiogenic response^6^.

Wound dressing materials are used as a protective barrier against pathogens during the wound healing process. These materials should be biocompatible. They should also help in cell attachment, proliferation, migration, and differentiation^3, 7^.

Hydrogels are insoluble hydrophilic matrices, which are proficient in absorbing huge amounts of water^8^. Hydrogels are capable of providing a moist environment and enhancing the autolytic debridement of necrotic tissues at the wound site^9^. Generally, dry wound dressings are ineffective in avoiding evaporation of water at the wound site, which ultimately results in reduced supply of nutrients to the new tissues^10^. Hydrogel dressings are flexible, rubbery, easily removable from the wound bed, and permeable to oxygen, water, and other nutrients^11^. Thus, they can easily transport enough gas and nutrients to the tissues^12^. Hydrogels are biocompatible and can improve cell attachment. In addition, they also mimic the extracellular matrix and are biodegradable against proteolytic enzymes^13^. These properties of the hydrogels help them to be used in tissue engineering and regenerative medical applications like diabetic wound healing.

Chitosan is a natural biopolymer that has been extensively used for the preparation of hydrogels, as it poses numerous desirable properties like good swelling, biocompatibility, low toxicity, and structural similarities with ECM^14^. Chitosan has been used as a base material with other polymers to prepare scaffolds, hydrogels, and films for several biomedical applications^15^.

Collagen is a structural protein essential for dermal reconstruction and tissue regeneration^16^. L-glutamic acid is a non-essential amino acid, which is a precursor for the synthesis of proline^17^. Proline and hydroxyproline are fundamental imino acids that are the major constituents in collagen structure^18^. An *in vitro* study revealed that the rate of collagen synthesis in granulation tissues depends on the concentration of proline and glutamic acid in the medium. Proline is synthesized from glutamic acid in granulation tissues during collagen synthesis^17^. Topical administration of L-proline showed a better wound closure rate during wound healing in rats^19^. In an earlier study by Tsao *et al*., chitosan/γ-poly(glutamic acid) polyelectrolyte complex was prepared and tested as a wound dressing material. This material showed suitable water uptake, mechanical strength, and helped in accelerated re-epithelialization^20^. In another study, the same group showed that the chitosan/γ-poly(glutamic acid) polyelectrolyte complex exhibited antibacterial activity, and was cytocompatible with NIH 3T3 fibroblast cells^21^. In another report, alginate, chitosan, and poly(γ-glutamic acid) were blended and prepared as hydrogels (AL-CS-PGA)^22^. This hydrogel was used as a dressing in type I diabetic rats for wound healing studies. The AL-CS-PGA hydrogel improved cell migration and accelerated epithelialization^22^. The results also showed that AL-CS-PGA hydrogel treatment increased the collagen content significantly in comparison to the alginate hydrogel^22^. Qualitative data obtained through immunohistochemistry analysis showed that loricrin was highly expressed in AL-CS-PGA hydrogel treated rats, indicating later differentiation of the epidermis^22^. In another work, an artificial dermis was fabricated by seeding adipose stem cells on poly(L-glutamic acid)/chitosan scaffolds^23^. The study showed that the artificial dermis treatment of wounds in type II diabetic mice resulted in improved angiogenic response, collagen deposition, and dermis thickness^23^. Recently, two different studies have prepared poly (γ-glutamic acid)-based hydrogels that were loaded with superoxide dismutase (SOD) and silk sericin^24, 25^. The SOD loaded hydrogel treatment showed increased collagen deposition and epithelialization in diabetic rats^24^. The silk sericin loaded hydrogel treatment showed improved cell proliferation and epithelialization in normal rats^25^. Although these studies have evaluated poly(glutamic acid)-based scaffolds, there are no reports on the potential of directly using L-glutamic acid for treating normal or diabetic wounds. As L-glutamic acid is a precursor for collagen synthesis and is significantly cheaper than poly(glutamic acid), we hypothesize that an effective wound dressing material can be developed by loading chitosan hydrogels with L-glutamic acid.

In this manuscript, we have reported the preparation of L-glutamic acid loaded chitosan hydrogel *via* physical crosslinking and have evaluated its potential in diabetic wound healing. These CS + LG hydrogels have been characterized for structural morphology, swelling, release, *in vitro* biodegradation, cytocompatibility, and *in vivo* diabetic wound healing.

## Results

### Hydrogel morphology

**Figure 1:**
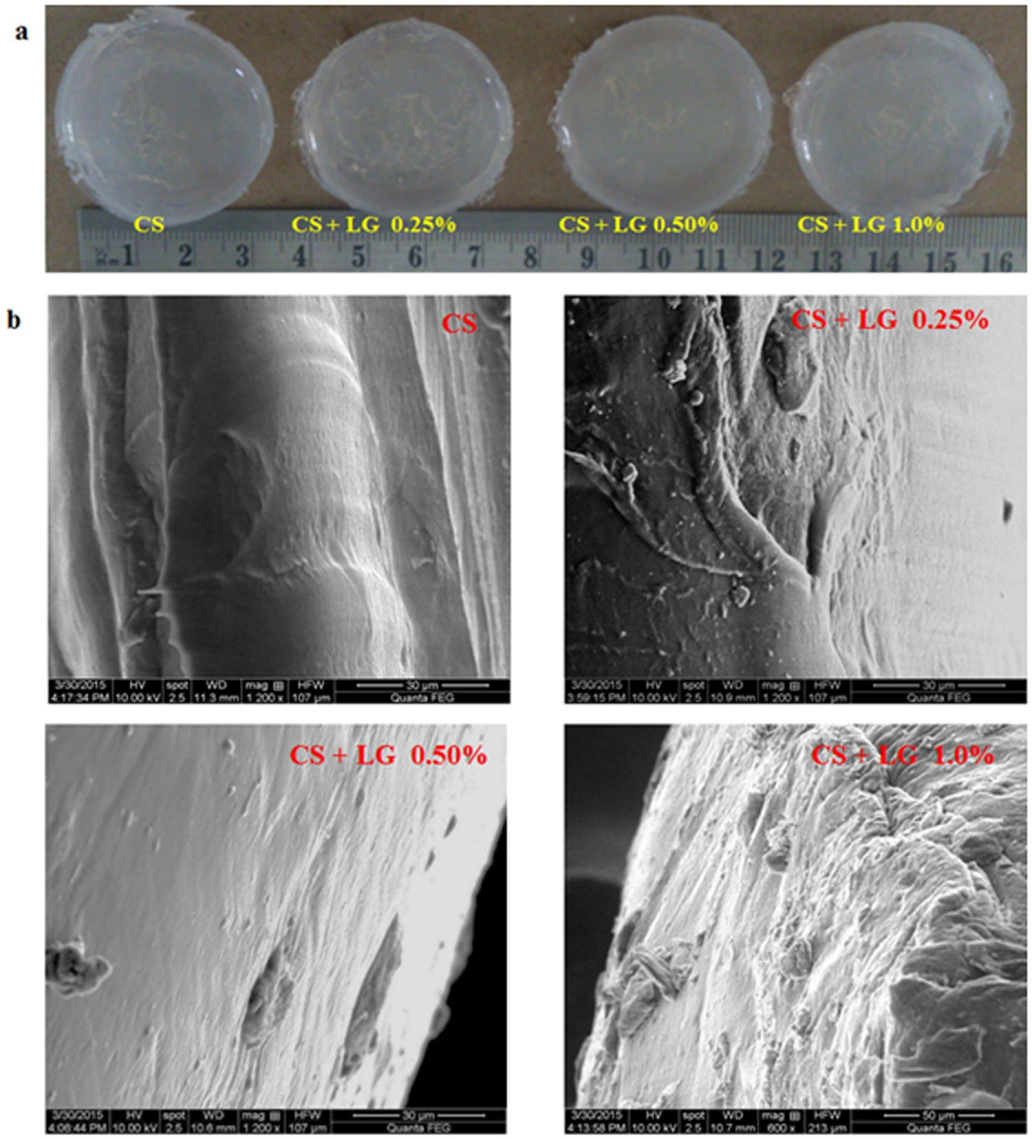
Morphology of the hydrogels. (**a**) Digital images of CS and CS + LG hydrogels (**b**) SEM images of CS and CS + LG hydrogels.

Digital images of the CS and CS + LG hydrogels are shown in Fig. 1a. Scanning electron microscopy analysis revealed the surface morphology of the hydrogels in Fig. 1b. CS hydrogels showed a plain smooth surface with a thin fibrous architecture. CS + LG (0.25 and 0.50%) hydrogels showed a slightly rough surface with a sponge-like filamentous structure. However, 1.0% LG incorporation showed rough architecture. Overall, CS + LG hydrogels exhibited rough architecture compared to CS hydrogels.

### Characterization of the hydrogels

**Figure 2:**
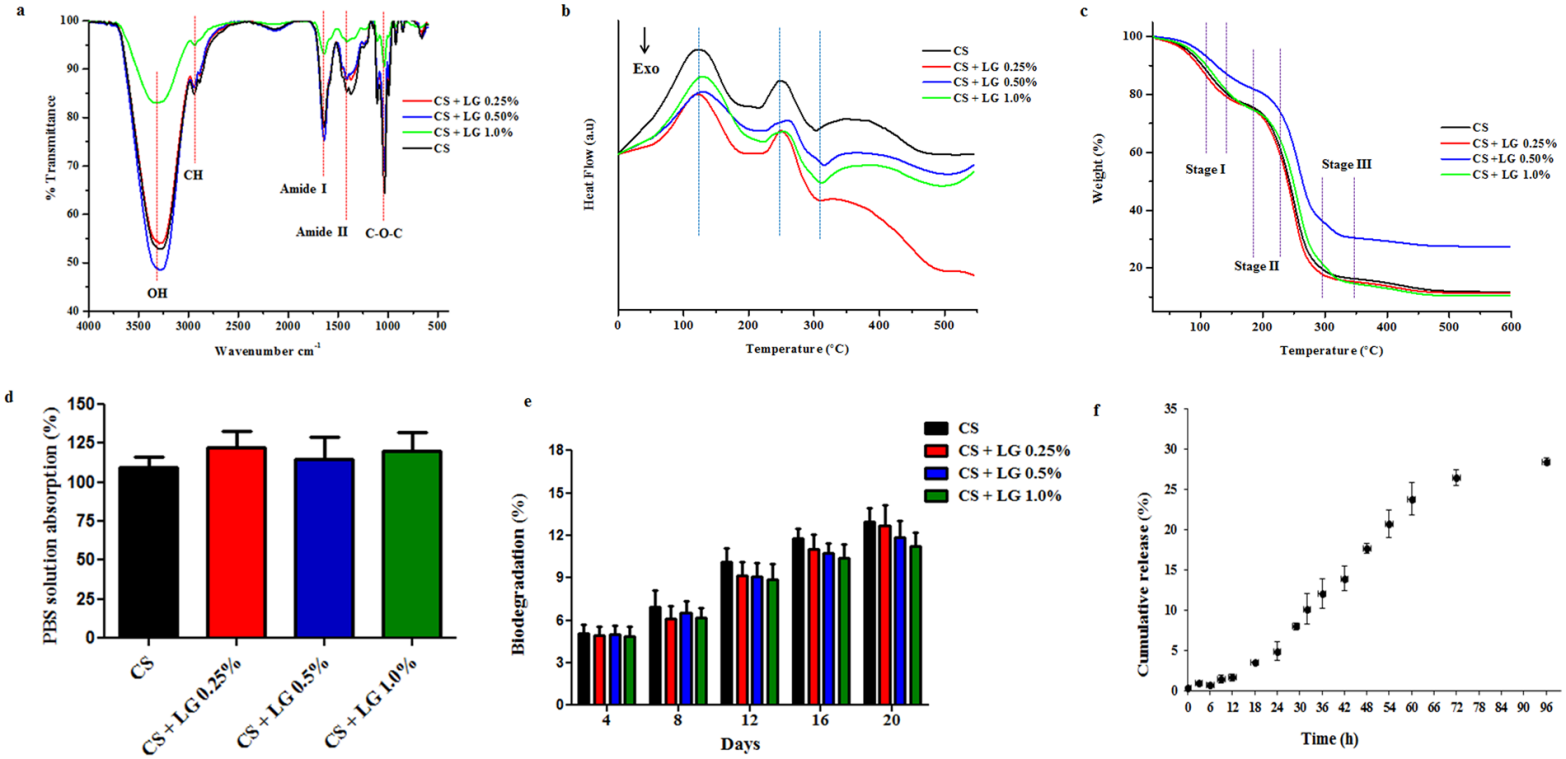
Characterization of the hydrogels. (**a**) ATR-FTIR spectra (**b**) DSC thermogram (**c**) TGA thermogram (**d**) PBS solution absorption (**e**) *In vitro* biodegradation (**f**) LG release profile. Values are stated as mean ± SD (n = 5).

CS and CS + LG hydrogels were characterized using ATR-FTIR spectroscopy. The spectrum is shown in Fig. 2a. The spectra showed a broad band around 3292 cm^−1^ for CS, and 3290–3341 cm^−1^ for CS + LG hydrogels. These peaks are attributed to the hydrogen bonding of internal water molecules. All of the hydrogels showed specific amide characteristic bands, viz., amide I (C=O stretching vibration, 1600–1650 cm^−1^), amide II (bending vibrations of the N-H bond, 1400–1450 cm^−1^), and amide III (N-H deformation, and C-N stretching vibrations, 1300–1250 cm^−1^)^8^. L-glutamic acid incorporation showed C-O-C asymmetric stretching at 1640 cm^−1^ and C-C stretching at 850 cm^−1^. The anti-symmetric stretching of C-O-C glycosidic bond observed at 1107–1110 cm^−1^ is associated with chitosan^26^. The band observed between 660–672 cm^−1^ is assigned to C=O in plane bending of COOH.

DSC spectra showed the first endothermic peak between 124 °C to 137 °C, corresponding to the removal of water molecules. A second endothermic peak was observed between 249 °C to 255 °C, which corresponds to the crystallization of the hydrogels. CS hydrogels showed the first endothermic peak at 124 °C representing the loss of water molecules and the second peak around 249 °C representing crystallization. L-glutamic acid incorporated chitosan hydrogels showed the endothermic peaks between 118 °C to 137 °C for water removal and 248 to 255 °C for crystallization. After crystallization, a strong degradation peak was observed around 310 °C for the CS and CS + LG hydrogels (Fig. 2b).

TGA analyses of the hydrogels showed three steps of sequential degradation (Fig. 2c). Pure chitosan hydrogels showed the first peak at 112 °C, with 24% weight loss associated with the removal of interlayer water molecules. The second thermal event for the decomposition of the polymer chain was noticed at 246 °C with a 24% weight loss. The third event, associated with carbonization of the hydrogel, was observed at 295 °C with a 16% weight loss. The residual mass was around 10%. CS + LG hydrogels showed comparable temperatures for all thermal events. The first thermal event was noticed between 107–124 °C for the removal of water molecules, with a 21–26% weight loss. The second thermal event occurred between 245–254 °C for the decomposition of the polymer chain with a 20–32% weight loss. The final thermal event was observed between 298–311 °C with a 4 to 29% weight loss. CS + LG hydrogels showed a residual mass of 7–12%.

### PBS solution absorption assay

The swelling property of the hydrogel regulates the supply of nutrients and removal of wastes. The swelling ratio is evaluated by the mass ratio of the hydrogel in the swollen state to that in the dry state^27^. Hydrogels were immersed in PBS solution (pH 7.4) at 37 °C to determine the swelling property. CS and CS + LG hydrogels absorbed over 100% of the dry weight within a short time. They reached equilibrium swelling within 1 h (Fig. 2d).

### *In vitro* biodegradation

Biodegradation is an essential property for the biomaterial. Biodegradation of the CS and CS + LG hydrogels were studied using 1 mg/mL lysozyme dissolved in PBS (pH 7.4) (Fig. 2e). Results showed that 11–13% of the degradation was achieved for all of the hydrogels following 20 days of incubation.

### Release profile

L-glutamic acid release from the CS + LG hydrogels was performed in PBS (pH 7.4) at 37 °C. In the first 12 h, only 2% of glutamic acid was released. At 24 h, 5% of LG had been released. At 72 h, it was observed that 27% of LG had been released. At the end of 96 h, the total release of LG was found to be 29% from the CS + LG hydrogels (Fig. 2f).

### *In vitro* biocompatibility of the hydrogels

**Figure 3:**
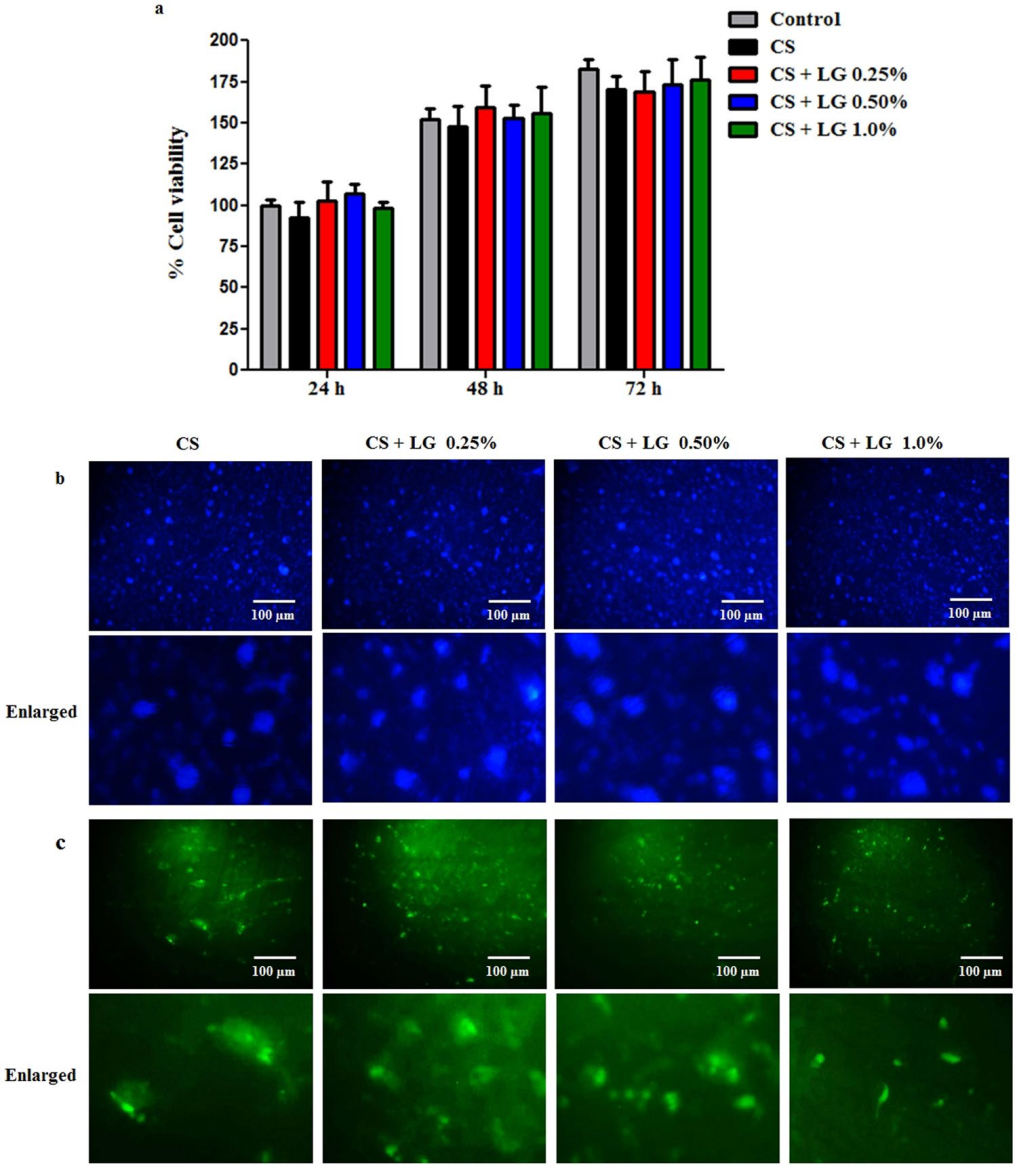
*In vitro* biocompatibility of the hydrogels. (**a**) NIH 3T3 cell viability at 24, 48 and 72 h. Values are stated as mean ± SD (n = 5). (**b**) Gross cell morphology of live and fixed cells using DAPI staining at 24 h and enlarged images shown (**c**) Cell proliferation by FDA staining at 24 h and enlarged images shown.

Biocompatibility was studied by culturing fibroblast cell lines (NIH 3T3) for 24, 48 and 72 h. The cell viability of the hydrogels was assessed by the alamar blue assay (Fig. 3a). Compared to control (tissue culture plate), CS and CS + LG hydrogels did not show any significant difference in cell viability. In addition, we used DAPI staining to identify the gross cell morphology of the live/fixed cells cultured on the hydrogels (Fig. 3b). DAPI nucleus staining showed that fibroblast cells absorbed DAPI solutions and were viable. CS + LG hydrogels (0.25% and 0.50%) showed similar trends for the cell attachment compared to CS hydrogels. Qualitative assessment of the DAPI stained images of CS + LG hydrogels (1%) showed a slight increase in the fibroblasts that were attached on the surface compared to the CS hydrogels. Image analysis of the DAPI stained images showed that the CS + LG hydrogels showed almost similar number of cells compared to the CS hydrogel.

FDA is a substrate for cell permeable esterase that evaluates the enzymatic activity and cell membrane integrity^28^. NIH 3T3 fibroblast cell proliferation and attachment on the hydrogel samples were observed by FDA staining. After 24 h, the proliferation of fibroblast cells on the hydrogel samples was examined under a fluorescence microscope (Fig. 3c). Qualitative assessment of the FDA stained images showed that fibroblasts were firmly attached and distributed on the CS + LG hydrogels. Image analysis of the FDA stained images showed that CS + LG 0.25% hydrogel showed slightly lower cell count compared to CS hydrogel. CS + LG 0.5% and CS + LG 1.0% hydrogels showed similar cell counts compared to CS hydrogel.

### *In vivo* wound healing in diabetic rats

Surgical open wounds were covered with CS and CS + LG hydrogel dressings and monitored for the wound contraction and re-epithelialization. *In vivo* results demonstrated that the use of CS and CS + LG hydrogels reduced the frequency of dressing and enhanced wound healing.

### Wound closure and period of epithelialization

**Figure 4:**
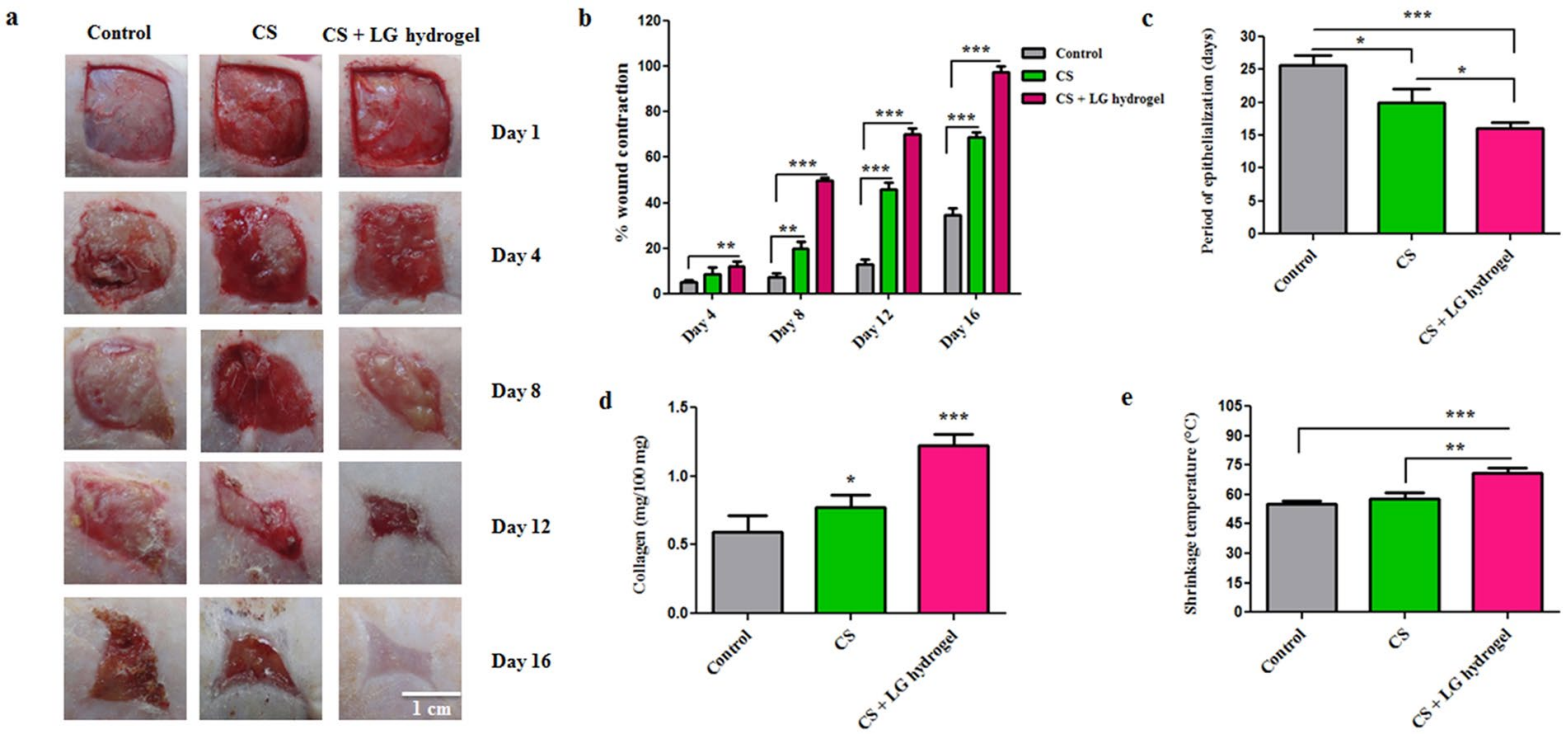
*In vivo* wound healing in diabetic Wistar rats. (**a**) Wound contraction images (Scale bar 1 cm) (**b**) Percentage wound contraction. (**c**) Period of epithelialization (**d**) Collagen content (**e**) Shrinkage temperature. Values are stated as mean ± SD (n = 5 rats) and the level of significance is denoted as *p < 0.05, **p < 0.01, and ***p < 0.001, respectively.

Wound closure was monitored in the diabetic rats and the wound contraction images are shown in Fig. 4a. The percentage wound closure in the diabetic rats was measured from day 0 to complete healing (Fig. 4b). Group I (control) showed around 5% wound contraction on day 4, 7% on day 8, 13% on day 12, and 34% on day 16. Diabetic rats treated with CS hydrogels showed a significant increase in wound contraction on days 4 (8%), 8 (20%), 12 (45%), and 16 (69%), compared to the control. Diabetic rats treated with CS + LG hydrogels showed wound contractions of 12% on day 4, 49% on day 8, and 70% on day 12, which were significantly higher (p < 0.001) compared to both control and CS hydrogel treatments. CS + LG hydrogel treated wounds achieved more than 97% wound contraction on day 16. However, control and CS hydrogel treated wounds took more time to achieve complete contraction. These results clearly indicate that CS + LG hydrogels could be a suitable material to treat chronic wounds.

Periods of epithelialization were also monitored (Fig. 4c). For complete healing, the diabetic control group showed around 26 days and CS hydrogel treatments showed 20 days (p < 0.05). However, CS + LG hydrogels took only 16 days (p < 0.001) for complete healing.

### Collagen content

Hydrogel dressings did not produce sufficient amount of granulation tissues on day 4. However, hydrogel dressings increased the rate of wound contraction superficially and produced less granulation tissue on day 8. Granulation tissues harvested from the wound site on day 8 were used to estimate hydroxyproline and the collagen in tissues (Fig. 4d). Granulation tissues of CS and CS + LG hydrogel treatments showed a significant increase in the amount of collagen on day 8. CS hydrogel dressings resulted in a 31% increase of collagen in the granulation tissues compared to control. CS + LG hydrogel treatments showed 106% and 57% increases in collagen content compared to control and CS hydrogel treatments, respectively.

### Shrinkage temperature

The shrinkage temperature measurement was performed on day 8 granulation tissue (Fig. 4e). CS hydrogel-treated granulation tissues showed 5% higher shrinkage temperature compared to control tissues. However, diabetic wounds treated with CS + LG hydrogels formed granulation tissues that had a significantly higher shrinkage temperature compared to control and CS hydrogels. The increases in shrinkage temperature of the granulation tissue from CS + LG treated diabetic wounds compared to control and CS hydrogels were 29% and 22%, respectively.

### Histopathology

**Figure 5:**
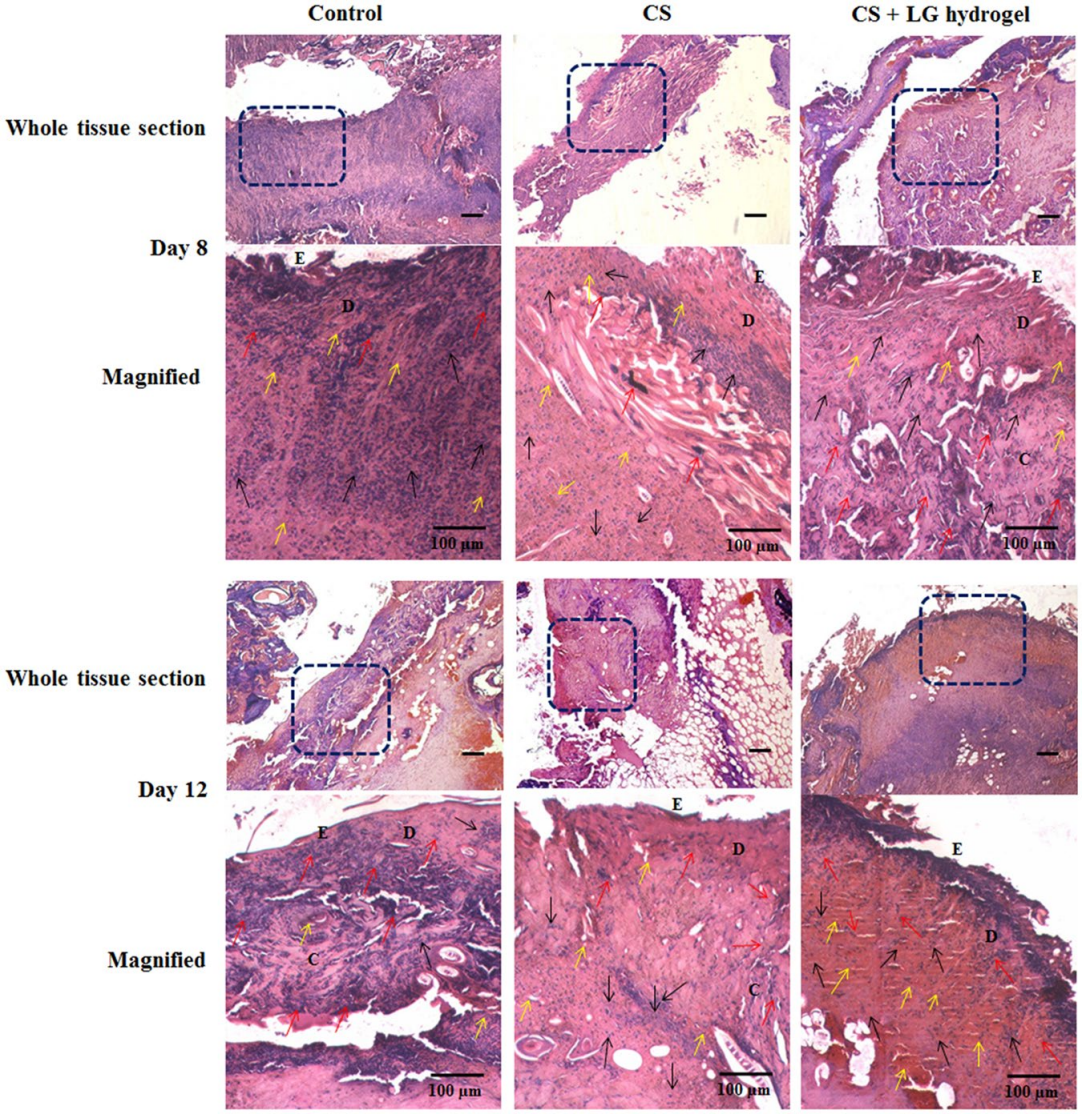
H&E staining. Histopathology results showing the hematoxylin and eosin (H&E) stained granulation tissues of days 8 and 12. The boxed regions in the whole tissue sections (4x; scale bar 25 µm) are displayed at higher magnification (20x; Scale bar 100 µm) as magnified images. CS+LG hydrogel treated diabetic wounds showed a thick epithelial layer, a large number of fibroblasts, collagen deposition, and abundant blood capillaries compared to control. Yellow arrows indicate the formation of blood vessels, red arrows indicate the fibroblast cells, and black arrows indicate the macrophages. E denotes the epidermis development and D denotes the dermis layer.

**Figure 6:**
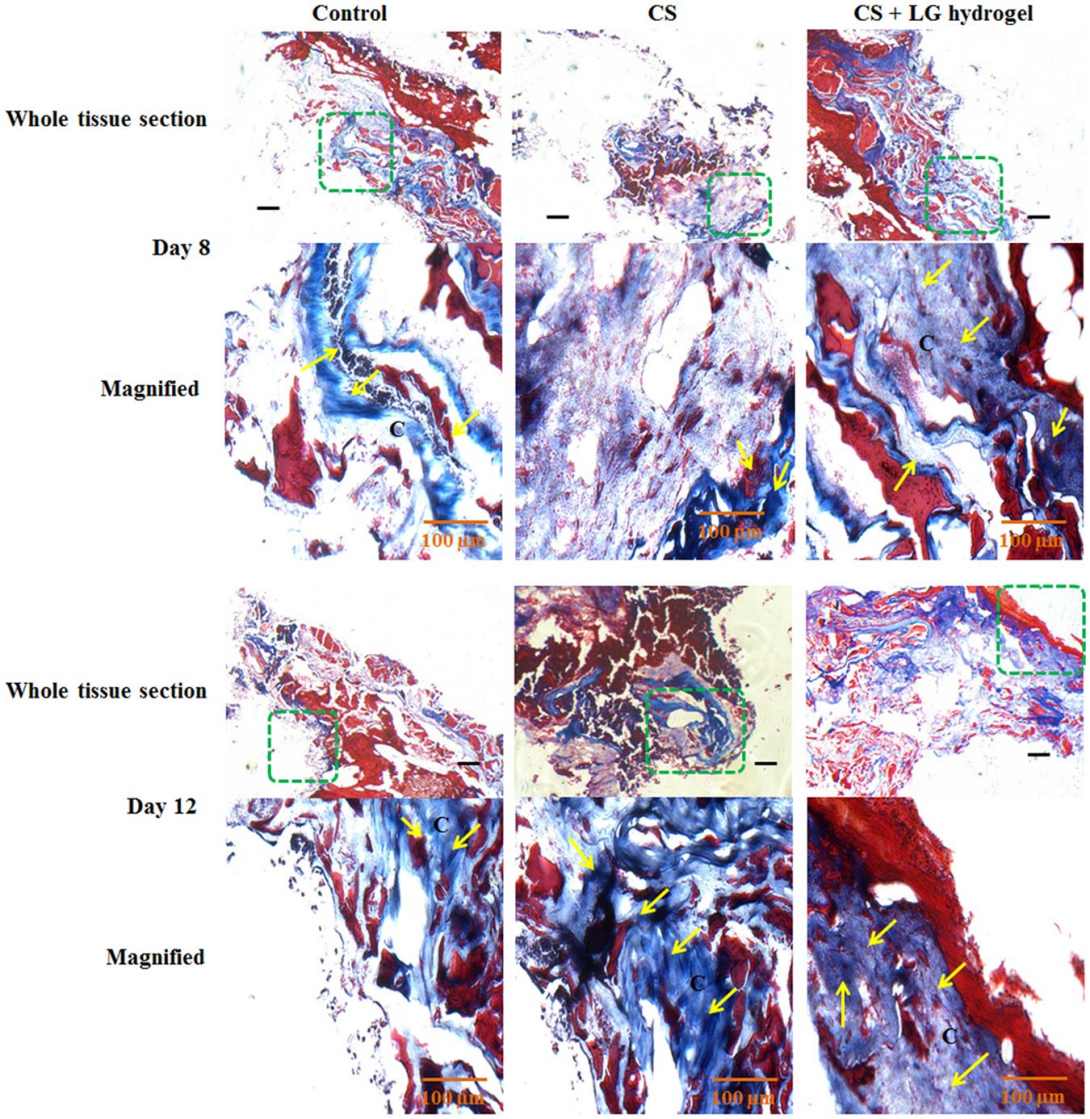
Masson’s trichrome staining. Synthesis and deposition of collagen were evaluated using Masson’s trichrome stained granulation tissues of days 8 and 12. The boxed regions in the whole tissue sections (4x; scale bar 25 µm) are displayed at higher magnification (20x; Scale bar 100 µm) as magnified images. CS+LG hydrogel treated tissues showed enhanced collagen synthesis, deposition, and maturation on days 8 and 12, compared to control and CS hydrogel treatment. Yellow arrows indicate the deposition of collagen bundles. C denotes the collagen fibers in treated tissues.

Microscopic analyses using histopathology led us to predict the event of wound healing in the granulation tissues collected on days 8 and 12. Tissue sections were stained with hematoxylin and eosin (H&E) and Masson’s trichrome stain. The early phases of wound healing and cellular activities were observed through H&E staining (Fig. 5). Collagen formation and deposition was qualitatively assessed using Masson’s trichrome staining (Fig. 6).

Dermal reconstruction can be evaluated by proliferation, remodeling, and maturation at the wound site. Diabetic control tissues showed prolonged inflammation and continuous infiltration of neutrophils and polymorphonuclear cells until day 8. However, day 12 control tissues showed fewer fibroblasts and blood vessels, as well as mild collagen deposition under the dermis layer. CS hydrogel-treated diabetic wounds displayed a thin epithelial layer and thick collagen bundles with lesser blood vessel formation on day 8 tissues. A prominent thick epithelial layer, huge blood vessels, macrophages, and more fibroblasts were found on day 12 treated tissues. CS + LG hydrogel-treated diabetic wounds showed more fibroblasts, increased collagen synthesis and deposition. A thick epithelial layer was observed on day 8 tissues. Day 12 tissues showed an abundance of red blood cells and a large number of blood capillaries, indicating an improved angiogenic process.

Masson’s trichrome stained tissues showed collagen deposition and its maturation in control, CS and CS + LG hydrogel-treated tissues on days 8 and 12 (Fig. 6). Control tissue showed lower amounts of collagen synthesis and deposition compared to CS and CS + LG hydrogel treatments. CS hydrogels increased the collagen synthesis and maturation compared to control. However, LG incorporation further elevated the rate of collagen synthesis and maturation compared to control and CS hydrogels.

### Immunohistochemistry

**Figure 7:**
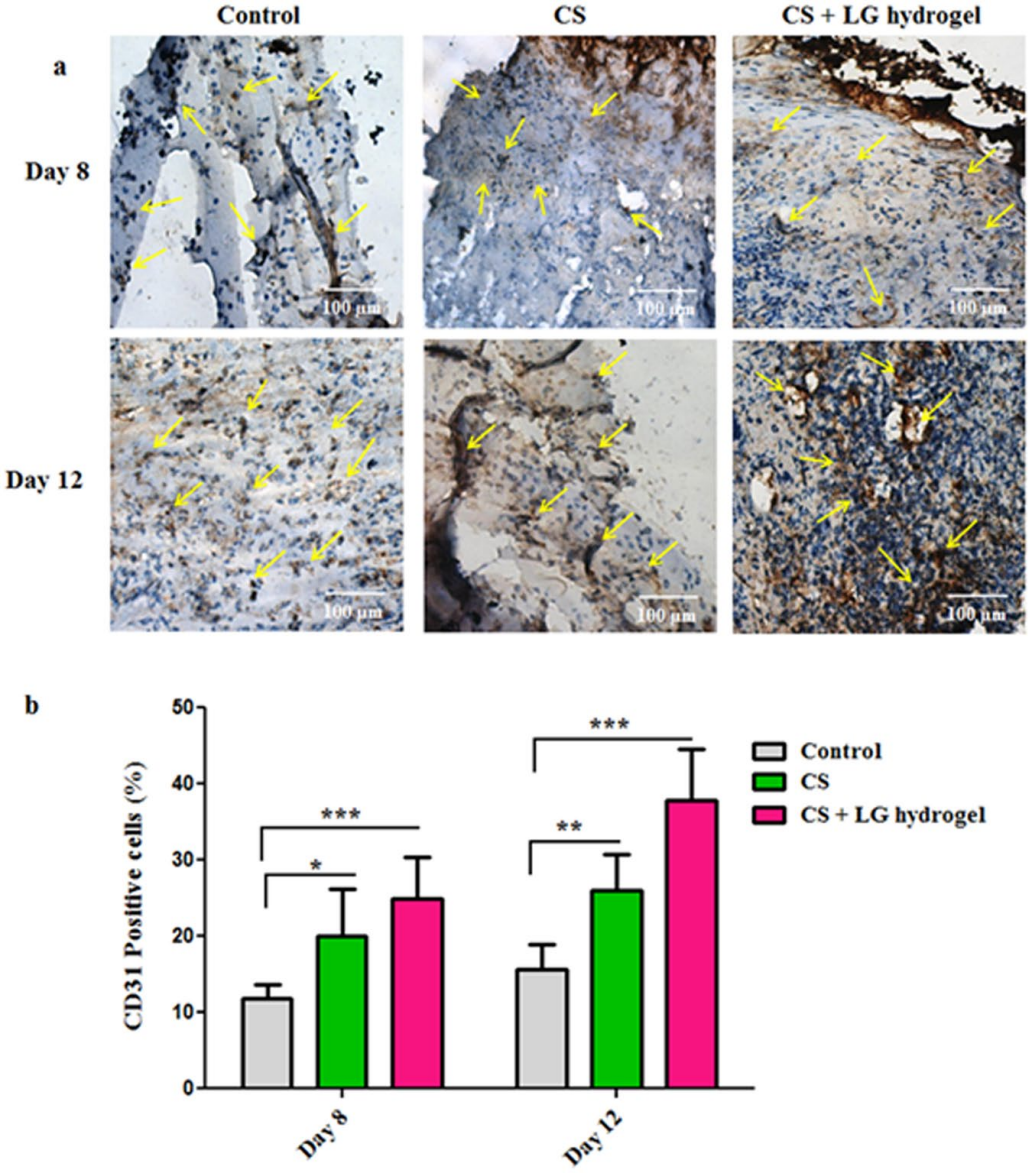
CD31 staining. (**a**) Immunohistochemistry results showing the CD31 stained granulation tissues of days 8 and 12. Yellow arrows indicate the blood vessels in control, CS and CS + LG hydrogel treated tissues. Scale bar 100 µm; magnification 20x. (**b**) Percentage of CD31-positive cells in granulation tissues of days 8 and 12. CS and CS + LG hydrogel treatment showed increased blood vessel formation. CS + LG hydrogel treatment showed a higher number of CD31-positive cells on days 8 and 12, with more functional vascular architecture. A higher number of newly formed blood vessels and more dense microvessels were also observed in day 12 granulation tissues from CS + LG hydrogel treated wounds, compared to control. Values are stated as mean ± SD (n = 5 rats), and the level of significance is conveyed as *p < 0.05, **p < 0.01, and ***p < 0.001 respectively, compared to the control.

Healing of diabetic wounds is delayed due to the improper angiogenic response and poor vascularization. CD31 staining was carried out in granulation tissues, obtained from the wounded area on day 8 and 12, to authenticate the newly formed blood vessels. The vascularity and blood vessel density of each tissue sections were analyzed and quantified (Fig. 7a,b). Controls showed a few immature blood vessels compared to CS and CS + LG hydrogel-treated wound beds on days 8 and 12. In comparison to controls, CS hydrogel treatment showed around 9% elevated CD31 positive cells on day 8 and 10% on day 12 (Fig. 7b). A few densely packed vessels were also observed. However, CS + LG hydrogel treatments showed a higher number of CD31 positive cells on days 8 and 12 with more functional vascular architecture. A higher microvascular density and more microvessels were observed on day 12, compared to control. CS + LG hydrogel-treated tissues exhibited a 15–22% increase in CD31 positive cells along with a higher vascular density on days 8 and 12 (Fig. 7b).

**Figure 8:**
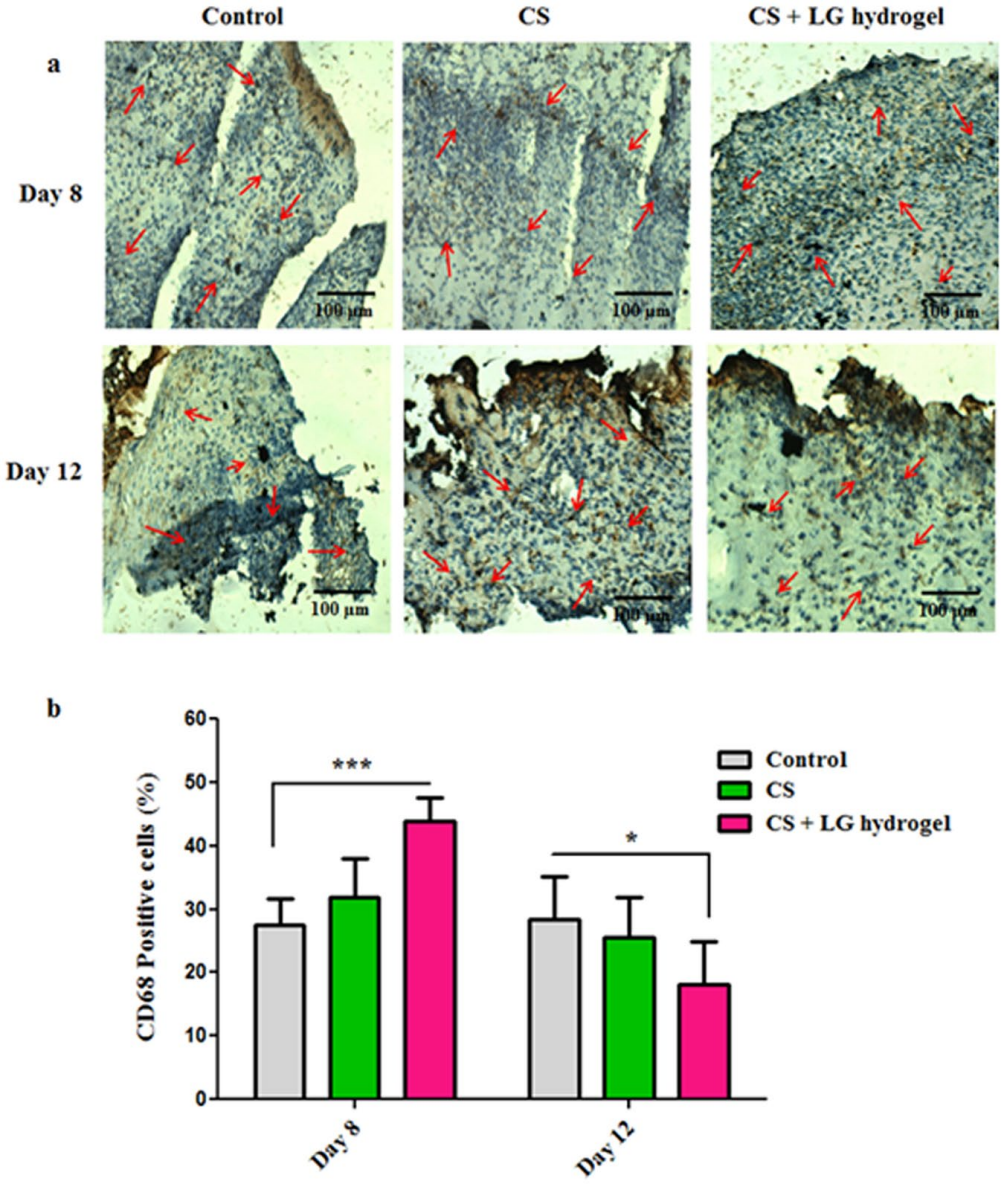
CD68 staining. (**a**) CD68 stained tissues showed the infiltration of macrophages/monocytes on days 8 and 12. Red arrows indicate the accumulation of macrophages/monocytes in control, CS and CS + LG hydrogel-treated tissues. Scale bar 100 µm; magnification 20x. (**b**) Percentage CD68-positive cells present in the granulation tissues of days 8 and 12. Significant elevation of CD68-positive cells on day 8 and reduction on day 12 suggest that CS and CS + LG hydrogels promote the diabetic wound repair process. Values are stated as mean ± SD (n = 5 rats) and the level of significance is denoted as *p < 0.05 and ***p < 0.001 respectively, compared to the control.

CD68 staining displayed the level of macrophages/monocytes in the tissue sections on days 8 and 12 (Fig. 8a). Results showed elevated levels of CD68 positive cells in CS and CS + LG hydrogel-treated tissues on day 8 compared to control. These results indicated that CS and CS + LG hydrogel dressings are probably involved in the early stage of inflammation and aid in recruiting the inflammatory cells like monocytes, macrophages, and neutrophils. However, the number of monocytes/macrophages were significantly (p < 0.05) reduced in CS + LG treated tissues on day 12, compared to control and CS hydrogel treatments (Fig. 8b). The reduction in CD68 cells on day 12 is a sign of the reduction in inflammation and active healing process in the CS + LG hydrogel treated diabetic wounds compared to control.

## Discussion

Diabetes is responsible for the delayed wound healing as a result of dysfunctional fibroblasts, epidermal cells, failed angiogenesis and tissue maturation^29^. Diabetes also causes decreased wound contraction and re-epithelialization during wound healing^30^. Hydrogel wound dressings have great attention as they provide enough moisture at the wound site to facilitate the cellular activities during wound healing^31^. In addition, they promise adequate flexibility, biodegradation, improved cell proliferation, attachment, faster wound contraction, neovascularization, and re-epithelialization^32^. Hydrogel dressings loaded with a bioactive molecule can have a great impact in accelerating diabetic wound healing.

In this work, we have developed chitosan based hydrogels loaded with L-glutamic acid to improve the diabetic wound healing ability of the hydrogel. SEM results showed a smooth surface for CS hydrogels and a fibrous architecture with sponge-like rough surfaces for the CS + LG hydrogels. This rough morphology can help in cell attachment and promote tissue repair at the wound site.

ATR-FTIR spectral characterization confirmed the presence of L-glutamic acid in the hydrogels. Results obtained from ATR-FTIR spectroscopy revealed that considerable interaction occurred between chitosan and L-glutamic acid. The deviation in the characteristic peaks was probably due to the incorporation of L-glutamic acid in chitosan. A shift in stretching vibrations was mainly observed in the amide, carbonyl and carboxyl regions of the chitosan and L-glutamic acid. DSC and TGA studies were used to analyze glass transition temperature, mass change, and thermal stability of the prepared hydrogels. The results clearly indicate that there was no structural deformation of chitosan due to L-glutamic acid incorporation.

When the CS and CS + LG hydrogels were immersed in PBS, they reached equilibrium swelling within one hour. All the hydrogels showed over 100% PBS absorption, which would provide a suitable moist environment for wound healing. *In vitro* biodegradation of these hydrogels were performed using 1 mg/mL of lysozyme in PBS. Lysozyme hydrolyzes the β (1–4) glycosidic linkage between D-glucosamine and N-acetyl D-glucosamine units of chitosan molecule at 37 °C, leading to the degradation of the hydrogels^33^. *In vitro* biodegradation results revealed that CS + LG hydrogels are biodegradable and can be used as a wound dressing material. The release profile of L-glutamic acid from the CS + LG hydrogels was very slow over the first 24 h. There was a sustained release of LG for the subsequent 48 h. However, our results indicate that the release of LG almost plateaus off after 72 h. A possible reason could be the low solubility of LG in the PBS. More studies are required to fully understand this release profile.

*In vitro* biocompatibility studies were performed by culturing NIH 3T3 cells on the control (tissue culture plate), CS and CS + LG hydrogels, for up to 72 h. CS and CS + LG hydrogels did not show any cytotoxicity, compared to control. As the results clearly indicate, the cells cultured on the CS and CS + LG hydrogels are viable and multiplying at a rate comparable to those on the control. Qualitative analysis of the DAPI and FDA stained images show that the cell attachment and proliferation are improved for the CS + LG hydrogels. Image analysis of DAPI and FDA stained images showed a similar number of cells were attached on CS and CS + LG hydrogels.

During the *in vivo* studies, it was observed that CS and CS + LG hydrogels showed good adhesiveness when applied topically at the wound surface. In addition, transparency of the hydrogels helped to monitor the wound closure regularly.

Wound contraction is a progressive process in the tissue matrix, which reduces the healing time by producing sufficient granulation tissue to repair the damaged tissue^34^. Re-epithelialization is a critical event in the wound healing process to regenerate and close the wound surface. Our study shows that CS + LG hydrogel-treated diabetic wounds (2 cm × 2 cm) showed complete epithelialization in 16 days. In an earlier study, AL-CS-PGA layered hydrogel dressings used in 1 cm × 1 cm wound models showed complete epithelialization in 15 days in female Wistar rats^22^. Another study showed that adipose stem cells seeded on poly(L-glutamic acid)/chitosan scaffolds resulted in 94% of epithelialization on the 14^th^ day in diabetic mice^23^. Compared to these reports, our results show a higher rate of epithelialization in diabetic rats. These findings clearly show that using L-glutamic acid helps in accelerated wound healing.

Collagen is the major component of extracellular matrix, which is essential for wound closure. Collagen synthesis, deposition, remodeling, and maturation are critical steps during tissue repair and regeneration^35^. Synthesis of collagen mainly depends on the availability of proline. Proline is actively synthesized in the granulation tissues from glutamic acid and the amount of proline synthesized is increased by lactate^17^. Increased collagen content observed in the CS + LG hydrogel-treated diabetic rats is most probably due to the availability of extracellular glutamic acid. Results indicate that loading of L-glutamic acid probably enhanced proline synthesis. Hence, an increase in collagen content is observed in the granulation tissues. Estimation of collagen and Masson’s trichrome staining confirmed that CS + LG hydrogel treatment increased the collagen content.

Shrinkage temperature, which represents the tensile strength of the granulation tissue, depends on the amount of hydroxyproline, proline, and inter-molecular crosslinking of the newly synthesized collagen^34^. Stability of collagen fiber is determined by the hydroxyproline concentration and its ability to crosslink. Hydroxyproline forms an ester crosslinking between polypeptide chains *via* inter-chain hydrogen bonding. This linkage mainly takes place between hydroxyl groups of hydroxyproline and carbonyl oxygen of the adjacent peptide^36^. Increased shrinkage temperature in CS + LG hydrogel-treated tissues indicates the deposition of a significant amount of crosslinked collagen at the wound site^37^. Overall results confirm that L-glutamic acid loaded hydrogels significantly increased the collagen content and crosslinking in diabetic wounds.

Angiogenesis or neovascularization is a key factor to restore the injured skin, and avoid later complications such as non-healing wounds and ulcers during diabetes. Defects in neovascularization, particularly in microvascular network formation, leads to interrupted tissue repair in diabetic conditions^38^. Acceleration of the neovascularization process in the early stage of the wound repair process is crucial for the regeneration of damaged tissues^39^. Immunohistochemistry (CD31) results indicate that CS + LG hydrogel treatments improve the angiogenic process by formation of blood capillaries and microvasculature, and maturation of blood vessels in diabetic wounds.

Prolonged inflammation, delayed recruitment of macrophages, and elevated neutrophils are reported for impaired wound healing in diabetes^40, 41^. CD68 staining shows the macrophage activity at the wound site. Infiltration of macrophages and neutrophils at the wound bed is part of the inflammation phase during wound healing^42^. Macrophages are the major inflammatory cells found at the wound site when the wound matures and neutrophils decrease^42^. Neutrophils are ingested by the macrophages at the wound site by the apoptotic process. Wound macrophages actively participate in the apoptosis process of neutrophils^43^.

Macrophage arrival at the wound site plays a vital role in skin repair. Studies revealed that macrophages play a variety of roles through their functional phenotypes during the wound repair process^42, 44^. Earlier studies reported that the reduced macrophage recruitment in the early inflammatory stage delays the granulation tissue formation and impairs re-epithelialization and wound contraction^44^. In addition, severe hemorrhage also occurs as a result of macrophage depletion during the early proliferative phase. Later, it prevents wound contraction and tissue maturation. However, decreased macrophage count in the maturation phase had no significant effect on wound closure^42^. In agreement with these findings, CS + LG hydrogel-treated diabetic wounds showed an elevated influx of macrophages at the early stage of wound healing and then decreased when compared to control. Overall, CS + LG hydrogel treatment accelerated wound contraction, epithelialization, angiogenesis, collagen synthesis and scar formation compared to control and CS hydrogel treatments in diabetic rats.

In conclusion, we have developed chitosan based hydrogels loaded with L-glutamic acid to improve the wound healing ability of the hydrogel. ATR-FTIR spectral characterization revealed the presence of L-glutamic acid in the hydrogels. SEM results showed smooth surfaces for CS hydrogels and fibrous architecture with a sponge like rough surfaces for the CS + LG hydrogels. These hydrogels showed desirable swelling properties and *in vitro* biodegradation. Compared to control and CS hydrogel treatments, topical dressings using LG-loaded hydrogels helped in faster wound healing in diabetic rats. L-glutamic acid loaded hydrogels reduced the frequency of wound dressing. Subsequently, it accelerated the cellular movements at the wound site for faster wound contraction and improved scar formation. These LG incorporated hydrogels reduced the epithelialization period and enhanced the rate of wound contraction in diabetic rats. It also increased the collagen content and crosslinking. Histopathological findings also revealed improved fibroplasia, collagen deposition, and angiogenesis. Immunohistochemistry results displayed that CS + LG hydrogel dressings improved the early angiogenic responses in treated wounds and reduced inflammation. Hence, CS + LG hydrogels could be an alternate wound dressing material for tissue repair and dermal reconstruction in chronic wounds.

## Materials and Methods

### Materials

Chitosan (molecular weight = 100–150 kDa, degree of deacetylation = 85%), lactic acid, L-glutamic acid, glycerol, and lysozyme were procured from Sigma Chemical Company, St. Louis, USA. All other reagents used were high analytical grade.

### Methods

All the methods were carried out in accordance with IIT Madras guidelines and regulations. All experimental protocols were approved by IIT Madras. Animal experiments were performed according to the protocols approved by the Institutional Animal Care and Use Committee of Central Leather Research Institute.

### Hydrogel preparation

Pure chitosan, chitosan + L-glutamic acid (25 mg, 50 mg, and 100 mg) 0.25–1.0% hydrogels were fabricated *via* physical crosslinking of 2 wt. % chitosan solution (in 2 wt. % lactic acid) and L-glutamic acid (in 1 mL, 1 M HCl). Glycerol (50 wt. %) was added to the above mixture, vortexed and poured into six well plates (Teflon molds). Previous literature has shown that glycerol and lactic acid are biocompatible and have been used with natural polymers to develop hydrogels^8, 45^. They provide adequate elasticity to hydrogels^45^. Later, these hydrogels were dried at 40 °C for 12 h. Then, the hydrogels were incubated in 1 M NaOH for 3 h. The excess NaOH in these hydrogels was washed away using distilled water and PBS^46^. These hydrogels were stored at room temperature for further studies (Fig. 1a).

### Characterization

Chemical characterization of the prepared hydrogels was performed using attenuated total reflectance-Fourier transform infrared spectroscopy (ATR-FTIR, Perkin Elmer). Differential scanning calorimetry and thermogravimetric analysis (DSC/TGA, NETZSCH STA 449 F3 Jupiter) were performed to study the glass transition temperature, thermal decomposition, and mass change of hydrogels. Scanning electron microscopy (SEM, Quanta 200) was used to assess the morphology of the hydrogels.

### PBS solution absorption assay

The swelling ability of the hydrogels was determined by immersing the hydrogels in phosphate buffered saline (PBS) solution (pH 7.4) at 37 °C for 1 h^47^. The PBS absorption ratio is defined as the ratio of weight increase (W_w_ − W_d_) to the initial weight (W_d_).

1 $${\rm{W}}=[({{\rm{W}}}_{{\rm{w}}}-{{\rm{W}}}_{{\rm{d}}})/{{\rm{W}}}_{{\rm{d}}}]\times 100$$

where W_w_ represents the wet weight of the hydrogels incubated in the PBS solution for 1 h and W_d_ is the initial weight of the dry hydrogels. The wet weight of the hydrogels (W_w_) was determined by weighing immediately after taking out from the PBS solution. The values are expressed as the mean ± standard deviation (n = 5).

### *In vitro* biodegradation

To understand the biodegradation of these hydrogels, they were incubated in PBS solution containing lysozyme, a proteolytic enzyme, for an extended period of time^48^. Hydrogels were cut into small pieces of almost equal size and weighed. The initial weight was noted as W_i_. These hydrogels were then submerged in PBS (pH 7.4) containing 1 mg/mL lysozyme and incubated at 37 °C for 20 days with a shaking speed of 100 rpm. On days 4, 8, 12, 16, and 20, the hydrogels were taken out and rinsed thoroughly with deionized water. These washed hydrogels were then lyophilized. The final weight of these hydrogels was noted as W_t_. The percentage biodegradation was calculated as:

2 $${\rm{Biodegradation}}\,( \% )=({{\rm{W}}}_{{\rm{i}}}-{{\rm{W}}}_{{\rm{t}}})/{{\rm{W}}}_{{\rm{i}}}\times 100$$

### L-glutamic acid release

L-glutamic acid release from hydrogels was determined by incubating hydrogel samples in 3 mL PBS solution at 37 °C. Aliquots were withdrawn at different time points and replaced with the same volume. The absorbance of these aliquots was measured at 270 nm using a UV-vis spectrophotometer (Jasco V-630). The L-glutamic acid concentration was estimated using a calibration curve prepared using L-glutamic acid solutions (R^2^ = 1.0)^8, 49^.

### Biocompatibility studies

Biocompatibility of the hydrogels was assessed in terms of percent cell viability of NIH 3T3 fibroblast cells using the alamar blue assay. The methods were carried out in accordance with the approved guidelines. Fibroblast cells were cultured in DMEM with 10% FBS supplemented with penicillin (100 units/mL), streptomycin (100 µg/mL), and amphotericin B (0.25 µg/mL) at 37 °C and humidified with 5% CO_2_. Sterile hydrogels were mounted in 12-well plates containing 5 × 10^4^ cells. The cell count was estimated based on the cell confluency using a standard chart. The cells were then incubated for 24, 48 and 72 h. The quantitative evaluation of cell viability and proliferation was performed using the alamar blue assay^50^. Briefly, alamar blue reagent (resazurin) was added to all the wells at a concentration of 0.1 mg/mL and incubated in 5% CO_2_ at 37 °C for 4 h. After the incubation, cell viability was quantified using absorbance measurements at 570 nm, with 600 nm as the reference wavelength^51^.

### Cell proliferation

The live and fixed fibroblast cells that were adsorbed on the hydrogel surface were stained using DAPI (4′,6′-di-amidino-2-phenyl-indol)^52^. Briefly, 5 × 10^4^ cells (NIH 3T3 fibroblasts) were seeded in each well of a 24-well tissue culture plate containing the hydrogels. These cells were left overnight for attachment. After 24 h of incubation, the culture medium was decanted from the wells. The cells were then rinsed thoroughly with PBS. Then, the cells were fixed for 10 min using 3.7% formaldehyde solution. These fixed cells were then washed thrice with PBS, to remove the formaldehyde. Then, cell permeabilization was performed in 0.2% Triton X-100 solution for 5 min. PBS was used to remove excess Triton X-100. DAPI stock solution (5 mg/mL) was diluted to 1:5000 in PBS. Cells were then incubated at room temperature for 5 min in DAPI staining solution. This solution was washed away using PBS. Later, hydrogels were observed for nuclei in five randomly chosen fields of vision at a magnification of 10x and 20x using an inverse fluorescence microscope (Olympus-IX 51). The digital micrographs were taken at an excitation wavelength of 359 nm^53^.

The cell morphology, proliferation, and attachment on hydrogel samples were observed using fluorescein diacetate (FDA) staining agent. The photomicrographs have been captured using inverse fluorescence microscope (Olympus-IX 51) with an excitation wavelength of 485 nm and an emission wavelength of 520 nm^54^. Image analysis of the DAPI and FDA stained images were performed using ImageJ software (Version 1.50i).

### *In vivo* wound healing in diabetic rats

Healthy male albino rats (Wistar strain) weighing between 150 and 180 g were used for the experiments. The rats were accommodated in wire topped cages with sterile rice husk as bed material. Rats were kept at temperatures between 22–25 °C under 12 h of light/dark cycles. Rats were fed with commercial rat feed (pellets) and water *ad libitum*. All the procedures were approved by the Institutional Animal Care and Use Committee of Central Leather Research Institute. A formal ethical approval was also obtained from the Institutional Animal Ethical Committee of Central Leather Research Institute prior to the animal experiments (IAEC No. 01/2015(b)/21.09.2015).

Single intraperitoneal injections of streptozotocin (STZ) and nicotinamide was used to induce experimental diabetes in overnight fasted rats. First, nicotinamide was dissolved in sterile water and injected at a concentration of 110 mg/kg body weight. After 15 minutes, STZ, dissolved in 0.1 M of cold citrate buffer (pH 4.5), was injected at a concentration of 50 mg/kg body weight^55^. After 72 h, diabetes was confirmed by measuring the tail vein blood glucose using a glucometer (Bayer Contour TS Blood Glucose Monitor). After three weeks of induction, rats with blood glucose level >250 mg dL^−1^ were considered as diabetic and used for the *in vivo* experiments.

The rats were divided into three groups, each group comprising five rats:

Group I: control rats, received gauze dressing;

Group II: dressed with pure chitosan (CS) hydrogel;

Group III: dressed with CS + LG 1.0% hydrogel;

### Surgical procedure and open excision wound creation

Thiopentone was dissolved in sterile water and administered intraperitoneally at a concentration of 50 mg/kg body weight to anesthetize the rats. Dorsal skin was shaved and then sanitized using ethanol. A 2 cm × 2 cm full thickness open excision wound was created surgically on the right side dorsal region of the rat^5^. Group I control wounds were dressed with sterile gauze. Groups II and III were covered with CS and CS + LG 1.0% hydrogel dressings, respectively. The dressing was replaced every four days until the wounds healed completely. The wound contraction was sketched on a tracing sheet and photographed. Granulation tissues formed on day 8 were collected and used for the biochemical analyses.

### Percentage wound contraction and period of epithelialization

Percentage wound contraction was estimated as the percentage of the reduction in the original wound size. The wound area was sketched on a transparent graph sheet, and the wound surface was measured planimetrically. Percentage wound contraction^19^ was calculated as:

3 $$ \% {\rm{Wound}}\,{\rm{contraction}}=\frac{{\rm{Wound}}\,{\rm{area}}\,{\rm{on}}\,{\rm{day}}\,0-{\rm{Wound}}\,{\rm{area}}\,{\rm{on}}\,{{\rm{n}}}^{{\rm{th}}}{\rm{day}}}{{\rm{Wound}}\,{\rm{area}}\,{\rm{on}}\,{\rm{day}}\,0}\times 100$$

where, n = number of days (Day 4, 8, 12 and 16).

During wound healing, the period of epithelialization was also measured. This represents the number of days taken for complete healing.

### Estimation of collagen

Granulation tissues collected on day 8 were used to estimate the collagen content^56^. Briefly, the fat layer in the tissue samples was removed using a 2:1 v/v mixture of chloroform and methanol. The defatted tissues were then frozen in acetone. The frozen tissue samples were weighed, hydrolyzed in 6 N HCl for 18 h at 110 °C, and dried. The treated samples were then made up to a known volume of double distilled water. From this, an aliquot was taken and spectrophotometrically at 557 nm^56^ to estimate the hydroxyproline content in granulation tissues. Assays were performed in triplicate and the collagen content was calculated as shown below^37^.

4 $${\rm{Collagen}}={\rm{Hydroxyproline}}\times 7.46$$

### Shrinkage temperature

Synthesis, deposition, and crosslinking of collagen fibers in granulation tissue are responsible for tensile strength and structural integrity of the tissue. Tensile strength was measured in day 8 granulation tissue in terms of shrinkage temperature^57^. Shrinkage temperature of the granulation tissues primarily depends on the strength and crosslinking of the newly synthesized collagen. Shrinkage temperature was measured using a shrinkage meter. Briefly, small pieces of granulation tissue samples were placed in a glass cavity slide and filled with 100 µL of water. Then, the slide was mounted on the top of a hot plate (heating stage) equipped with a tungsten lamp. This hot plate was connected with a thermometer and an optical microscope. Then, the tissue samples were heated at a constant rate. The temperature at which the sample shrinks was considered the shrinkage temperature.

### Histological analyses

The granulation tissues collected on days 8 and 12 were used for the histopathological analysis. Tissue samples were fixed in 10% formalin–saline, dehydrated in graded alcohol series, cleared in xylene and embedded in paraffin wax. Serial sections (5 μm thickness) were cut and mounted on glass slides. Then, the tissue sections were stained with hematoxylin & eosin (H&E), and Masson’s trichrome stains. Stained tissue sections were examined under a light microscope (Olympus-IX 51) for the histological analyses.

### Immunostaining and analyses

Formalin saline fixed 5 μm tissue sections were first deparaffinized using xylene and then dehydrated with graded ethanol series. The slides were boiled in 10 mM citrate buffer (pH 6.0) for 30 min to retrieve the antigens required for detecting CD31 and CD68 positive cells. Nonspecific protein-binding sites were blocked using antibody diluent (Dako S2022) with normal goat serum (Dako, Glostrup, Denmark). Then, tissue sections were incubated overnight with primary antibodies, mouse monoclonal antibodies against CD31 (PECAM1; Dako) and CD68 (PGM1; Dako). After blocking endogenous peroxidases and washing with PBS, the sections were treated with peroxidase-labeled polymer conjugated to goat anti-mouse immunoglobulins A (Dako). The sections were decolorized, counterstained with hematoxylin, and analyzed for the vascular density and macrophage/monocyte positive cells on days 8 and 12 granulation tissues. For the quantification of CD31-positive vessels and CD68-positive macrophages, five images were taken from three tissue sections and analyzed using ImageJ IHC profiler (Version 1.50i). The percentage of CD31-positive blood vessels and CD68-positive cells were quantified as a percent score using ImageJ IHC profiler^58, 59^.

### Statistics

All the values are expressed as the mean ± standard deviation (n = 5) and the results obtained were analyzed using Student’s t-test. Statistical analyses were performed using GraphPad Prism (version 5.0; GraphPad software Inc. San Diego CA, California, USA). The values of *p < 0.05, **p < 0.01, and ***p < 0.001 were considered as statistically significant.
